# Placental growth factor may predict increased left ventricular mass index in patients with mild to moderate chronic kidney disease – a prospective observational study

**DOI:** 10.1186/1471-2369-14-142

**Published:** 2013-07-11

**Authors:** Martina Peiskerová, Marta Kalousová, Vilem Danzig, Blanka Míková, Magdalena Hodková, Eduard Němeček, Amjad Bani-Hani, David Ambrož, Hana Benáková, Ales Linhart, Tomas Zima, Vladimir Tesař

**Affiliations:** 1 Department of Nephrology, First Faculty of Medicine, Charles University, Prague, Czech Republic; 2 Institute of Medical Biochemistry and Laboratory Medicine, First Faculty of Medicine, Charles University and General University Hospital, Prague, Czech Republic; 3 2nd Department of Medicine - Department of Cardiovascular Medicine First Faculty of Medicine, Charles University and General University Hospital, Prague, Czech Republic; 4 Clinical Biochemistry, Haematology and Immunology, Na Homolce Hospital, Prague, Czech Republic

**Keywords:** Cardiovascular disease, Chronic kidney disease, Echocardiography, Extracellular newly identified RAGE-binding protein (EN-RAGE), Left ventricular mass index, Left ventricular hypertrophy, Left ventricular diastolic function, Placental growth factor (PlGF)

## Abstract

**Background:**

Placental growth factor [PlGF) is a cardiovascular (CV) risk marker, which is related to left ventricle hypertrophy (LVH) in animal models. Currently there are no data available regarding the possible relationship of PlGF and the development of LVH or diastolic dysfunction in patients with chronic kidney disease (CKD) and the relationship of PlGF to other CV risk factors in CKD patients. The aim of our study was to determine the possible association of PlGF and several other CV risk markers to echocardiographic parameters in CKD population.

**Methods:**

We prospectively examined selected laboratory (PlGF, fibroblast growth factor-23 -FGF23, vitamin D, parathyroid hormone, extracellular newly identified RAGE-binding protein - EN-RAGE, B-type natriuretic peptide - BNP) and echocardiographic parameters in 62 patients with CKD 2–4. Mean follow-up was 36 ±10 months. Laboratory and echocardiographic data were collected 2–3 times, at the shortest interval of 12 months apart. Multivariate regression analysis was used to detect independent correlations of variables.

**Results:**

Increased left ventricular mass index (LVMI, g/m^2.7^) was found in 29% patients with CKD 2–4, left ventricular (LV) diastolic dysfunction was detected in 74.1% patients (impaired LV relaxation in 43.5% patients and pseudonormal pattern in 30.6% patients). After 36 ± 10 months increased LVMI was found in 37.1% patients with CKD 2–4, LV diastolic dysfunction was detected in 75.8% patients (impaired LV relaxation in 43.5% patients and pseudonormal pattern in 32.3% patients). Following independent correlations were found: LVMI was related to PlGF, cholesterol, BNP, systolic blood pressure and serum creatinine. EN-RAGE correlated positively with left atrial diameter and inversely with E/A ratio. During the follow-up we found a significant increase in LVMI and left atrial diameter, whereas a significant decrease in LVEF was noted.

**Conclusion:**

According to our data, PlGF is independently related to increased LV mass in CKD, whereas EN-RAGE is more likely related to diastolic dysfunction in this population.

## Background

Cardiovascular risk in patients with chronic kidney disease is increased in early stages of renal insufficiency and rises with its progression. Traditional as well as specific CKD-related risk factors lead to vascular calcification, left ventricular hypertrophy (LVH) and myocardial fibrosis [1-3]. In CKD patients, LVH is a common condition originating in early CKD stages and its prevalence progresses with declining renal function [4]. LVH may develop as a compensatory mechanism to volume and pressure overload, but finally it contributes to the unfavourable outcome. LVH in CKD is typically accompanied by collagen accumulation, arteriolar wall thickening, calcification, and capillary rarefaction, reduction in the number of cardiomyocytes and hypertrophy. These mechanisms accelerate the onset of systolic and diastolic dysfunction of the left ventricle. Left ventricular (LV) diastolic dysfunction is an abnormality of relaxation, filling or distensibility of the left ventricle that portends a poor prognosis regardless of any associated systolic dysfunction [5]. Three types of LV diastolic dysfunction involve: 1. impaired relaxation (grade I) 2. pseudonormalization (grade II) and 3.restrictive filling (grade III).

A number of pathways possibly responsible for the high CV risk in CKD are currently being studied. These mechanisms include hypertension, hyperactivity of the renin-angiotensin-aldosterone system, anaemia, sodium and volume retention, endothelial dysfunction, mineral and vitamin D disorders, micro-inflammation and oxidative stress [3]. These pathways are under constant research, including investigation of biomarkers possibly linking CKD to CV pathology, such as placental growth factor (PlGF), extracellular newly identified RAGE-binding protein (EN-RAGE), metalloproteinases, fibroblast growth factor 23 (FGF23), 25OHvitaminD and parathyroid hormone (PTH).

One of the above mentioned biomarkers - Placental growth factor (PlGF) - is a 149 aminoacid heterodimer, expressed in human placenta, heart, thyroid gland, lung and skeletal muscle. PlGF is a member of the pro-proliferative vascular endothelial growth factor family and a pro-atherogenic cytokine which stimulates angiogenesis in ischemic tissues. It is up-regulated in atherosclerotic lesions, stimulates vascular smooth muscle growth and up-regulates production of tumour necrosis factor (TNF). PIGF is a biomarker of vascular inflammation and CV risk [6]. In animal models, PlGF is related to LV hypertrophy [7,8], however little is known about the relation of PlGF to LVH in human population. Another pro-atherogenic molecule, Pregnancy associated protein (PAPP-A), belongs to the family of metalloproteinases (MMPs). It has been found in plasma, vascular smooth muscle cells and in atherosclerotic plaques. High plasma levels of PAPP-A have been found in dialysis patients [9]. Products of non-enzymatic glycation and oxidation of proteins and lipids, advanced glycation-end products (AGEs), accumulate in CKD and they play a role in the development of atherosclerosis. Binding of AGEs to their receptor (RAGE) activates the pro-inflammatory transcription factor NF-kB. EN-RAGE is an extracellular ligand for RAGE which has been found to exert pro-inflammatory effects [10]. Impaired calcium-phosphate metabolism is another factor contributing to the high CV morbidity and mortality in CKD [11] and vitamin D deficiency resulting in increased plasma FGF23 levels in CKD patients might directly cause vascular calcification, increased arterial stiffness, endothelial dysfunction and LV hypertrophy [12].

No data exist so far, about the possible relationship of PlGF and the development of LVH or diastolic dysfunction in CKD patients and the possible relationship of PlGF and other CV risk markers. Little is known about echocardiographic changes in patients with earlier CKD stages. Therefore, we aimed to study the possible association of PlGF and several other pro-atherogenic molecules or CV risk markers with echocardiographic parameters in CKD 2–4 patients.

## Methods

Between December 2004 and May 2009, 76 subjects with mild to moderate renal insufficiency (CKD 2–4) were consecutively recruited in the Outpatient unit of the Department of Nephrology (General University Hospital, Charles University, Prague). These subjects were followed during a mean period of 36 ± 10 months. We prospectively examined selected laboratory and echocardiographic characteristics of these subjects. Data were collected 2–3 times, at the shortest interval of 12 months apart. During the follow up period 8 patients died and 6 withdrew the informed consent. Final data analysis was performed only in 62 patients who completed the whole follow up period. Estimated glomerular filtration rate (eGFR) was calculated by MDRD formula. CKD was defined as a reduction in eGFR below 1 ml/s/1.73 m^2^.

Clinical and demographic characteristics of the group are presented in Table 1. Etiology of CKD was: ischemic nephropathy (21%), IgA nephritis (15%), chronic pyelonephritis (13%), hypertensive nephropathy (11%), diabetic nephropathy (10%), ANCA associated vasculitis (5%), lupus nephritis (5%), and other (20%). About 92% of patients received ACE inhibitors and/or AR blockers, 13% were substituted with calcium, 44% received calcitriol and 61% were on statin therapy.

**Table 1:** Baseline clinical and demographic characteristics of the study group

**Variable ± SD**	
**Number of patients**	62
**Age (years)**	62 ± 15
**Men**	37
**Women**	25
**BMI (kg/m**^**2**^**)**	26,9 ± 3,9
**Hypertension %**	88,7
**Mean systolic BP (mm Hg)**	133 ± 16
**Mean diastolic BP (mm Hg)**	80 ± 7
**Number of antihypertensive drugs**	3 ± 2
**History of CVD %**	50
**DM %**	21

*Abbreviations*: *BMI* Body mass index, *BP* blood pressure, *CVD* cardiovascular disease, *DM* Diabetes mellitus.

*History of CV disease* was taken from medical records of each patient, comprising coronary heart disease, peripheral arterial obstructive disease and/or cerebrovascular disease. History of CV disease was noted in 31 patients (50%). No patient had symptoms of severe heart failure (NYHA III. or IV.) or hemodynamically significant valvular defect.

### Blood samples

Fasting venous blood samples from each patient were collected. All samples were centrifuged for 10 min at 1.450 g (4°C). Sera were stored at −80°C until analysis.

### Biochemical analysis

FGF23 (C terminal fragment) was measured with ELISA kit according to the manufacturer protocol (Immune topics, San Clements, CA, USA). PAPP-A was assessed immunochemically with the TRACE (Time Resolved Amplified Cryptate Emission) technology based on non-radiating energy transfer (commercial kit KRYPTOR-PAPP-A, Brahms, Germany). MMP-2 and PlGF were measured with ELISA, Standard kits Quantikine, RD systems, Minneapolis, MN, USA. Biointact parathyroid hormone levels were analysed with ECLIA method (ROCHE, analyser MODULAR SWA). Brain natriuretic peptide (BNP) and troponin I (cTnI) were measured by chemiluminiscence methods (UniCel DxC 880i - Beckman Coulter analyzer). sRAGE and EN-RAGE were measured using standard ELISA kits according to the manufacturers’ protocols: sRAGE (Quantikine, RD Systems, Minneapolis, MN, USA, http://www.rndsystems.com), EN-RAGE (CirculexTM, CycLex Co. Ltd., Nagano, Japan, http://www.cyclex.xo.jp). Routine biochemical parameters were assessed by standard laboratory methods.

Echocardiography was carried out approximately 2 hours after blood sampling.

Complete two-dimensional M-mode and Doppler studies were performed via standard approaches, using Vivid 7 (GE Medical system, Waukesha, Winconsin). M-mode examination was performed according to American Society of Echocardiography guidelines [13] LV mass was determined using standard formula, as follows: Left ventricular mass = 0.8 × (1.04 × (LVEDD + PWTd + SWTd)^3^ – (LVEDD)^3^) + 0.6 [13]. The values were indexed by the patient’s height^2.7^, thus obtaining left ventricular mass index (LVMI). LV hypertrophy was defined as LV mass index >46.7 g/m^2.7^ in women or 49.2 g/m^2.7^ in men. Relative wall thickness, calculated as 2-times posterior wall thickness divided by LV internal diastolic dimension, was used to characterise LV geometry into following categories: normal (≤ 0.42 and normal LVM), concentric remodeling (normal LVMI but RWT > 0.42), concentric hypertrophy (− increased LVMI and RWT > 0.42), and eccentric hypertrophy (− increased LVMI and RWT ≤ 0.42). LV volumes, comprising end-diastolic (LVEDV) and end-systolic volume (LVESV) were estimated using modified Simpson method, and used to calculate LV ejection fraction. Doppler characteristics of LV filling and diastolic function were assessed by using transmitral flow pattern along with pulmonary venous inflow parameters. In most patients we recorded mitral annular velocities. According to the current recommendations the filling was categorized as normal, impaired relaxation, pseudonormal and restrictive [14,15]. Left atrial diameter (LAD) was indexed to body surface area, obtaining the parameter LAD/BSA (mm/m^2^). In patients with mild diastolic dysfunction, the mitral E/A ratio is < 0.8, deceleration time of inflow of the E wave, (DT) is > 200 ms. In patients with moderate diastolic dysfunction (grade II), the mitral E/A ratio is 0.8 to 1.5 (pseudonormal) and decreases by ≥ 50% during the Valsalva maneuver. With severe diastolic dysfunction (grade III), restrictive LV filling occurs with an E/A ratio ≥ 2, DT < 160 ms [14,15].

The study was approved by the Ethical Committee of General University Hospital in Prague, reference number: 50/08. A written informed consent was obtained from all participants.

### Statistics

The results of biochemical parameters are expressed as mean ± SD, in case of non-normal data distribution as medians and interquartile ranges. Comparisons were conducted with paired sample t tests for normally distributed continuous variables and Wilcoxon test for non-normal distributions. Variables with non-normal distributions were ln- transformed where appropriate. Association among analyzed parameters was assessed by Pearson’s correlation coefficient. Subsequently, linear regression analysis for determinants of echocardiographic parameters influential variables was performed. All variables significantly associated with echocardiographic characteristics were included in the multiple regression stepwise analyses (serum albumin, PlGF, serum cholesterol, 25OH vitamin D, BNP, FGF23, serum creatinine, EN-RAGE, PTH, PAPP, Pi, sRAGE, serum TAG, MMP2). Qualitative variables, such as tobacco smoking, history of CV disease, use of ACE inhibitors, were analysed using the Kruskal-Wallis test. Chi-Squared Test for Trend was used to compare baseline and final echocardiographic findings in the subject group (Table 2). Results were considered as statistically significant at p < 0.05. All analyses were performed using MedCalc 9.3 (MedCalc Software Comp. Mariakerke, Belgium).

**Table 2:** Echocardiographic characteristics (%) of the study group (n = 62)

	**Baseline**	**After 36 months + − 10**	**p value chi square test for trend**
**LV mass index (g/m**^**2.7**^**)**			
normal	71,0	62,9	p = 0.22 NS
increased	29,0	37,1	
**LV geometry**			
normal LV geometry	56,5	43,5	p = 0.25 NS
concentric remodelation	12,9	21,0	
concentric hypertrophy	9,7	9,7	
excentric hypertrophy	21,0	25,8	
**LVEF (%)**			
normal	88,7	87,1	p = 0.68 NS
decreased	11,3	12,9	
**LAD (cm/m**^**2**^**)**			
normal	98,4	98,4	p = 1.00 NS
increased	1,6	1,6	
**LV diastolic function**			
normal LV diastolic function	25,8	24,2	p = 0.96 NS
impaired relaxation	43,5	43,5	
pseudonormal pattern	30,6	32,3	
**E/A ratio**			
below 0.8	46,8	48,4	p = 0,06 NS
0-8-1.5	50,0	40,3	
above 2	3,2	11,3	
**DTE-MI (ms)**			
above 200	38,7	62,9	p < 0.01
160-200	37,1	27,4	
under 160	24,2	9,7	

*Abbreviations*: *E/A ratio* Ratio between early (E) and late (*atrial* - A) ventricular filling velocit, *DTE-MI* Decelaration Time on Mitral Valve, *LAD* left atrial diameter, *LV* left ventricular.

## Results

1. **Baseline echocardiographic parameters of the study group (Table**2**).**

Increased LV mass was noted in 29% patients. We identified 56.5% subjects with normal LV geometry, 12.9% subjects with concentric remodelling, 9.7% subjects with concentric hypertrophy and 21% subjects with eccentric hypertrophy. Normal LV diastolic function was found in 25.8% patients, impaired LV relaxation in 43.5% patients and pseudonormal pattern in 30.6% patients. No one met the criteria of restrictive pattern of LV diastolic filling.

2. **Echocardiographic parameters of the study group after 36 ± 10 months (Table**2**).**

Increased LV mass was noted in 37.1% patients. We identified 43.5% subjects with normal LV geometry, 21% subjects with concentric remodelling, 9.7% subjects with concentric hypertrophy and 25.6% subjects with eccentric hypertrophy. Normal LV diastolic function was found in 24.2% patients, impaired LV relaxation in 43.5% patients and pseudonormal pattern in 32.3% patients. No one met the criteria of restrictive pattern of LV diastolic filling.

**Figure 1:**
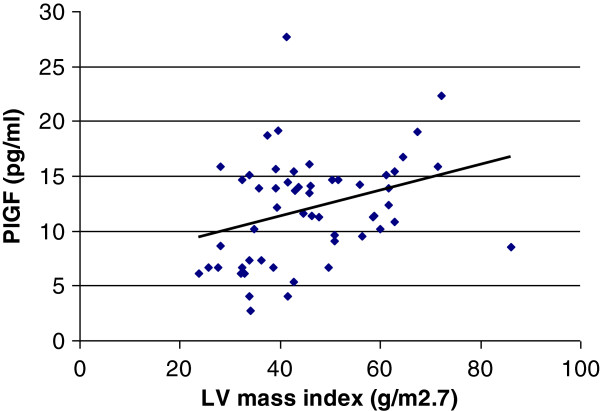
**Correlation of PlGF levels to LV mass index (g/m**^**2.7**^**) r = 0.31, p < 0.02.** Based on final data. PlGF: placental growth factor, LV: left ventricle.

3. **Independent correlations of echocardiographic parameters, laboratory markers and blood pressure (Table**3**, Figure**1**).**

**Table 3:** Independent correlations of laboratory and echocardiographic parameters (stepwise multiple regression)

	**LVMI1**	**LVMI3**	**LAD 1**	**LAD 2**	**LAD 3**	**EF1**	**EF2**	**EF3**	**E/A 1**	**E/A 2**	**E/A 3**
**MDRD**	r = −0,31	r = −0,37	r = −0,25	***r = −0,37***	***r = −0,41***	-	-	-	***r = 0,54***	***r = 0,43***	***r = 0,40***
	p = 0,02	p < 0,01	p = 0,06	***p < 0,02***	***p < 0,01***				***p < 0,0001***	***p < 0,01***	***p < 0,01***
**Serum Albumine**	r = −0,27	-	-	***r = −0,33***	r = −0,33	-	-	-		-	-
	p < 0,05			***p < 0,05***	p = 0,02						
**PTH**	-	-	-	-	-	-	***r = −0,47***	-	-	-	-
							***p < 0,01***				
**EN-RAGE**	-	-	***r = 0,35***	-	-	r = −0,26	-	-	***r = −0,34***	-	-
			***p < 0,01***			p < 0,05			***p = 0,01***		
**PIGF**	-	***r = 0,31***	-	-	r = 0,36	-	-	-	-	-	-
	-	***p < 0,02***			p < 0,01						
**BNP**	***r = 0,42***	***r = 0,51***	r = 0,27	r = 0,30	***r = 0,50***	-	-	-	-	-	-
	***p < 0,01***	***p < 0,001***	p < 0,05	p = 0,08	***p < 0,01***						
**systolic BP**	-	r = 0,31	-	-	-	-	-	-	-	-	-
		p < 0,02									

Legend: The 3 values for each parameter stand for serial echo exams at different time points (1: baseline assessment, 2: control 1 assessment 3: control 2 assessment). Only significant correlations are presented, independent correlations are highlighted.

*Abbreviations*: *BNP* brain natriuretic peptide, *BP* blood pressure, *E/A* Ratio between early (E) and late (*atrial* - A) ventricular filling velocity, *EF* left ventricular ejection fraction, *EN-RAGE* Extracellular newly identified RAGE-binding protein, *DT* deceleration time on mitral valve, *LAD* left atrial diameter, *LVMI* left ventricle mass index, *MDRD* modification of diet in renal disease, *PlGF* placental growth factor, *PTH* parathyroid hormone, *r* Pearson correlation coefficient.

LV mass index was positively related to PlGF, BNP, systolic BP and serum creatinine. BNP positively correlated also with left atrial diameter. EN-RAGE was positively related to left atrial diameter and inversely to E/A. PTH inversely correlated with LVEF. No independent correlations were found between echocardiographic parameters and haemoglobin, PAPP-A, FGF23 or vitamin D levels. PlGF was not related to blood pressure.

**Figure 2:**
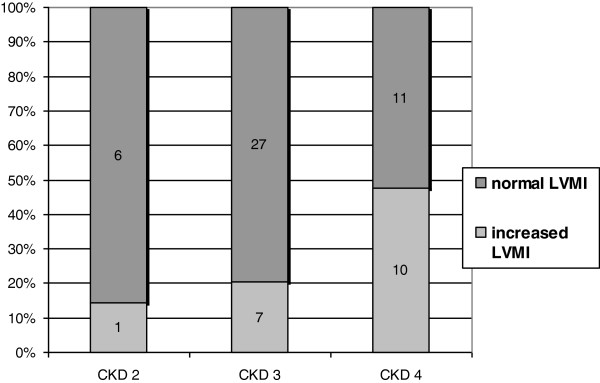
**LV mass index (g/m**^**2.7**^**) in different CKD stages.** Based on baseline data. LV: left ventricle, CKD: chronic kidney disease.

4. **Impact of renal function on laboratory and echocardiographic parameters and their changes during the follow-up period (multiple regression) (Table**4**, Figure**2**).**

**Table 4:** Changes of laboratory and echocardiographic parameters during the follow-up period

**Parameter**	**Baseline**	**After 18 months ± 5**	**After 36 months ± 10**	***p value for Baseline vs. 18 months assessment***	***p value for 18 months assessment vs. 36 months assessment***	***p value for Baseline vs. 36 assessment***
**eGFR (MDRD) (ml/s)**	0.6	0.57	0.49	p < 0.01	p < 0.05	p < 0.01
	(0.25-1.6)	(0.25-1.3)	(0.26-2.8)			
**Haemoglobin (g/l)**	128.5 ± 20.0	128.8 ± 20.7	124.3 ± 18.8	p < 0.01	p < 0.01	NS
**Serum Phosphate (mmol/l)**	1.10	1.16	1.20	NS	NS	NS
	(1.00-1.29)	(1.00-1.45)	(1.00-1.36)			
**Parathyroid hormone (pg/ml)**	5.96	6.34	7.52	NS	NS	NS
	(3.56-9.22)	(4.56-11.98)	(3.63-15.59)			
**25OH Vitamin D (ng/ml)**	23.47 ±8.91	25.04 ±9.61	20.87 ±7.79	NS	p < 0.01	p < 0.02
**FGF23 (RU/ml)**	89.6	100.1	127.0	p < 0.01	p < 0.05	p < 0.0001
	(64.8-167.1)	(73.0-228.8)	(78.3-282.4)			
**PAPP-A (mIU/l)**	8.3	8.7	9.3	NS	NS	NS
	(7.0-10.2)	(7.6-10.5)	(7.5-12.6)			
**sRAGE (pg/ml)**	976.3	919.1	1040.7	NS	NS	NS
	(720.6-1495.2)	(643.9-1336.3)	(719.1-1375.1)			
**EN-RAGE (ng/ml)**	160.5	255.2	269.8	p < 0.05	NS	p < 0.001
	(100.5-240.3)	(164.6-297.0)	(163.0-326.3)			
**PlGF (pg/ml)**	10.80	11.05	12.5	p < 0.02	NS	NS
	(7.8-14.2)	(8.5-15.6)	(8.5-14.7)			
**MMP-2 (ng/ml)**	214.5 ±50.6	206.5 ±39.2	221.9 ±61.6	NS	NS	NS
**Troponin I (ng/ml)**	0.01	0.01	0.01	NS	NS	NS
	(0.01-0.01)	(0.01-0.01)	(0.01-0.01)			
**BNP (pg/ml)**	30.0	57.0	77.0	p < 0.01	NS	p < 0.0001
	(15.0-91.0)	(27.8-107.3)	(40.0-195.0)			
**Left ventricle mass index (g/m**^**2.7**^**)**	43.6 ± 14.6	45.3 ± 16.0	45.7 ± 13.4	NS	NS	p < 0.05
**Left ventricle EF (%)**	64.7 ± 7.8	64.5 ± 5.8	62.7 ± 8.0	p < 0.05	NS	p < 0.05
**Left atrial diameter (cm/m**^**2**^**)**	2.14 ± 0.64	2.05 ± 0.55	2.19 ± 0.50	NS	NS	p < 0.01
**E/A ratio**	0.83	0.83	0.81	NS	NS	NS
	(0.67 - 1.14)	(0.69 - 0.98)	(0.72-1.04)			

Arithmetic mean ± SD or median (Interquartile Range).

Comparison: Paired samples *t*-test for normal distributions, resp. Wilcoxon test for skewed distrubutions.

*Abbreviations*: *BNP* brain natriuretic peptide, *BSA* body surface area, *E/A ratio* Ratio between early (E) and late (*atrial* - A) ventricular filling velocity eGFR: estimated glomerular filtration rate, *EN-RAGE* Extracellular newly identified RAGE-binding protein, *FGF23* fibroblast growth factor 23, *MDRD* modification of diet in renal disease, *MMP-2* matrix-metaloproteinase 2, *PAPP-A* pregnancy associated protein A, *PlGF* placental growth factor.

eGFR was positively related to E/A ratio and inversely related to LVMI and left atrial diameter. During the follow-up, with the decline of eGFR, we noted a significant increase in LVMI, left atrial diameter, EN-RAGE, FGF23 and BNP, whereas a decrease was observed in LVEF, serum albumin, vitamin D and haemoglobin. No significant changes in blood pressure were noted.

5. **Laboratory parameters in patients with history of CV disease.**

History of CV disease was noted in 50% of patients. These patients had higher LVMI (p < 0.02), serum creatinine (p < 0.01), triacyglycerols (p < 0.05), FGF23 (p < 0.02) and PAPP-A (p < 0.05), whereas they had lower 25OHvitamin D (p < 0.05) and serum albumin levels (p < 0.01), compared to those free of such history. Relation of PlGF to CV disease history was of borderline significance (p = 0.05).

To sum up the results: During the follow-up period (initially, resp. after 36 ± 10 months) we noted an increased LVMI in 29%, resp. 37.1% of patients, abnormal LV geometry in 43.5%, resp. 56.5% and abnormal LV diastolic function in 74.1%, resp. 75.8 of subjects. However, these trends were not significant. LVMI correlated with PlGF, BNP, systolic BP and eGFR. LV diastolic function was related to EN-RAGE and eGFR. During the follow-up, with declining eGFR we noted an increase in LVMI, left atrial diameter, EN-RAGE, FGF23 and BNP, whereas a decrease was observed in LVEF, serum albumin, vitamin D and haemoglobin.

## Discussion

In the group of patients with mild to moderate CKD, we noted a high prevalence of LV remodelling and increased LV mass with rising frequency in more severe CKD. We detected increased LVMI in 14% patients with CKD 2, in 21% with CKD 3 and in 48% patients in CKD 4 stages (Figure 2, Table 2). Levin et al. have reported the prevalence of LVH in 26.7% of patients with GFR > 50 mL/min, in 30.8% of those with GFR between 25 and 49 mL/min and in 45.2% of patients with severe CKD (GFR <25 mL/min [16], which is more or less in accordance with our findings. High prevalence of increased LVMI in CKD has been repeatedly described [16-18], but the studies are difficult to compare due to different definitions of LVH, different study populations and variations in blood pressure control, including the use of ACE inhibitors and/or ARBs.

LV mass index in our study correlated independently with systolic BP, BNP, serum creatinine and PlGF. The relationship of BNP to LVMI and CV pathology has already been described [19-21] and a correlation of LVMI to BNP, CRP and troponin T has been reported in CKD 3–4 stages [22]. However, in our present study we failed to show a significant correlation of LVMI to troponin or CRP.

In contrast, the correlation of PlGF to LV mass in CKD patients has not been reported so far. In an animal study [23], PlGF overexpressing mice exhibited a greater cardiac hypertrophic response, an increase in capillary density and in fibroblast content in the heart in response to stress. Thus, PlGF overexpressing mice showed a type of cardiac growth, which was protective against signs of failure. Contrarily, PlGF(−/−) mice died of heart failure within 1 week of pressure overload. PlGF probably works through endothelial cells and fibroblasts, secondarily stimulating the myocytes through paracrine factors, such as interleukin-6 [24]. PlGF has been reported to stimulate angiogenesis in the infarction border [7], to promote atherosclerotic intimal thickening and macrophage accumulation and has been associated with long-term prediction of CHD [25,26]. In human atherosclerotic lesions PlGF expression has been associated with plaque inflammation, suggesting its role in plaque destabilization. Delivery of anti-PlGF antibody delayed the progression of atherosclerotic plaques to vulnerable lesions [24]. The expression of PlGF in human vascular endothelial and smooth muscle cells has been reported to be induced by angiotensin II and aldosterone [8] and conversely, mineralocorticoid antagonists have been shown to inhibit PlGF expression in human vessels [27]. PlGF levels have been reported to be significantly higher in CKD 1–4 stage and hemodialysis patients compared to controls [28]. Increased PlGF levels have been found in patients with CV event history compared to those free of such history [28] which is in accordance with our findings.

LV hypertrophy is a strong predictor of CV events and of the risk of progression to dialysis [18]. Interestingly, several studies in CKD patients have shown the lack of correlation between blood pressure and LV mass [29], suggesting that other neuro-humoral factors, promoting myocardial fibrosis, might play a key role in the increase of LV mass. In CKD patients, LV mass has been reported to be independently related e.g. to albuminuria, FGF23 [29-31] and to CaxPO4 [32]. In a prospective study in 3879 CKD subjects, higher FGF23 levels were associated to mortality risk [12]. FGF23 caused hypertrophy of isolated rat cardiomyocytes and treatment with an FGF-receptor blocker attenuated LVH. According to our data, FGF-23 correlated with LVMI, however this relation did not prove independent in multiple regression analysis. In contrast, we noted an independent inverse correlation of PTH levels with LVEF. Association of PTH with myocardial hypertrophy, fibrosis and higher coronary lesion score was described in animal model [33].

LV diastolic dysfunction has been observed already in CKD 1–2 stages [15,33]. CKD severity was the most independent predictor of elevated LV filling pressure [34,35]. Our baseline data in CKD 2–4 show normal diastolic function in 25.8% in of patients, impaired relaxation in 43.5%, and pseudonormal pattern in 30.6% of subjects (Table 2).

We noted a positive correlation of EN-RAGE with left atrial diameter and an inverse correlation with E/A. The RAGE pathway could be a causal risk factor for LVH and coronary atherosclerosis. Recent data show that EN-RAGE (also called S100A12) contributes to inflammation and atherosclerosis [36] and an early blockade of RAGE by statins may prevent inflammation in atherosclerosis [37]. S100A12 levels have not been reported to be elevated in CKD patients, but they have been shown to be positively correlated with CRP and negatively correlated with sRAGE [28]. An inverse relationship has been described between sRAGE and LVMI in CKD patients [38,39], but in the present study we failed to note such a correlation.

During the follow-up period we noted a rising percentage of subjects with increased LVMI, abnormal LV geometry, decreased LVEF and LV diastolic dysfunction (Table 2), but this trend was not significant, probably due to the time span limited to 36 ± 10 months.

Currently, the regression of LVH may be achieved mainly by antihypertensive and anemia treatment [16,40]. Of note, 48 week therapy with paricalcitol did not alter LVMI or improve diastolic dysfunction in patients with CKD (PRIMO study) [41]. To specifically target LVH in the CKD population, we need to better understand the molecular events that promote LVH even in the absence of pressure or volume changes in CKD. Randomized controlled trials are needed to find whether LVH, cardiac fibrosis, and electrical instability that plague patients with CKD can be prevented by aggressive multifactorial therapy started early in CKD, possibly including therapeutic lowering of PlGF, FGF23 or EN-RAGE levels. In this prospective observational study we performed repeated laboratory assessment in a close timely relation to echocardiographic measurements, in order to analyse dynamic changes and correlations of these parameters. We must call attention to some limitations of the present study: due to a relatively high number of variables and statistical tests performed in a limited number of subjects, we cannot exclude the possibility of false positive findings. However, appropriate multiple regression stepwise analyses (i.e. a multimarker approach) to detect independent correlations of variables, were performed. We did not consider appropriate to perform ROC curves, as this analysis is considered meaningful in at least 100 observations [42]. Another limitation is the assessment of the filling pattern only from transmitral flow. However, normal pattern was distinguished from pseudonormal by experienced cardiologists taking into account also pulmonary venous flow, left atrial dilatation and in some patients also tissue Doppler imaging. We did not systematically perform the mitral annulus excursion velocity measurements using tissue Doppler, since it was not routinely used in 2005, at the beginning of the study.

## Conclusions

Our data describe for the first time an independent and significant relationship of PlGF to increased LV mass in mild to moderate CKD. EN-RAGE seems more likely related to diastolic dysfunction in this population. We report serial echocardiographic changes in CKD 2–4, such as increased LV mass index and diastolic dysfunction progressing with time along with the declining renal function. Further investigation is needed to demonstrate which strategy is most efficient in preserving the cardiac structure and function.

## Abbreviations

ACE: Angiotensin-converting-enzyme; AGEs: Advanced glycation Endproducts; ANCA: Anti neutrophile cytoplasma antibodies; ARBs: Angiotensin II receptor; BNP: B-type natriuretic peptide; BSA: Body Surfaře Area; CKD: Chronic kidney disease; cTnI: Cardiac Troponin I; CV: Cardiovascular; DT: Decelaration Time; DTE-MI: Decelaration Time on Mitral Valve; E/A ratio: Ratio between early (E) and late (*atrial* - A) ventricular filling velocity; ECLIA: Electrochemiluminescence immunoassay; eGFR: Estimated glomerular filtration rate; ELISA: Enzyme-Linked ImmunoSorbent Assay; EN-RAGE: Extracellular newly identified RAGE-binding protein; FGF-23: Fibroblast growth factor 23; IgA: Immunoglobuline A; IVSd: Interventricular Septal Thickness at Diastole; LAD: Left atrial diameter; LV: Left ventricle; LVEDD: Left ventricular end-diastolic diameter; LVEDV: Left ventricular end-diastolic volume; LVEF: Left ventricular ejection fraction; LVESV: Left ventricular end-systolic volume; LVH: Left ventrikle hypertrophy; LVMI: Left ventricular mass index; MDRD: Modification of. Diet in Renal Disease; MMPs: Matrixmetalloproteinases; ms: millisecond; NF-kB: Nuclear factor kappa-light-chain-enhancer of activated B cells; NYHA: New York Heart Association; PAPP-A: Pregnancy associated protein; PlGF: Placental growth factor; PRIMO: study Paricalcitol Capsules Benefits in Renal Failure Induced Cardiac Morbidity in Subjects With Chronic Kidney Disease Stage 3/4; PTH: Parathyroid hormone; PWTd: Diastolic posterior wall thickness; RAGE: Receptor for Advanced glycation Endproducts; ROC: Receiver operating characteristic; RWT: Relative wall thickness; SWTd: Diastolic septal wall thickness; TAG: Triacylglycerols; TNF: Tumour necrosis factor.

## Competing interests

All the authors declare that they have no competing interests.

## Authors’ contribution

MP participated in sample collection, clinical data collection, laboratory processing and preparation of manuscript. MK is the main consultant, took part in laboratory processing, interpretation of the data and preparation of manuscript. MH was inestimable in sample collection and clinical data collection. VD, EN, AB and DA are experienced cardiologists who effected echocardiographic measurements, interpretation of the data and manuscript preparation. BM was responsible for statistical analysis. HB participated in biochemical analysis of study samples. TZ, AL and VT provided expert opinion, took important part in data interpretation and manuscript preparation. All authors read and approved the final manuscript.

## Pre-publication history

The pre-publication history for this paper can be accessed here:

http://www.biomedcentral.com/1471-2369/14/142/prepub
